# The Aging-Related Prognostic Signature Reveals the Landscape of the Tumor Immune Microenvironment in Head and Neck Squamous Cell Carcinoma

**DOI:** 10.3389/fonc.2022.857994

**Published:** 2022-05-10

**Authors:** Fang Chen, Xin Gong, Meng Xia, Feng Yu, Jian Wu, Chaosheng Yu, Junzheng Li

**Affiliations:** ^1^ Department of Otorhinolaryngology-Head and Neck Surgery, Zhujiang Hospital, Southern Medical University, Guangzhou, China; ^2^ Department of Otolaryngology, Head and Neck Surgery, Wushan County People’s Hospital of Chongqing, Chongqing, China; ^3^ Department of Otorhinolaryngology-Head and Neck Surgery, Guangzhou Red Cross Hospital of Jinan University, Guangzhou, China

**Keywords:** aging-related genes, prognosis, tumor immune microenvironment, immune checkpoint, head and neck squamous cell carcinoma (HNSCC)

## Abstract

**Background:**

Numerous studies have shown that the aging microenvironment played a huge impact on tumor progression. However, the clinical prognostic value of aging-related risk signatures and their effects on the tumor immune microenvironment (TIME) in head and neck squamous cell carcinoma (HNSCC) remains largely unclear. This study aimed to identify novel prognostic signatures based on aging-related genes (AGs) and reveal the landscape of the TIME in HNSCC.

**Methods:**

Differentially expressed AGs were identified using the gene set enrichment analysis (GSEA). The prognostic risk model of AGs was established by univariate and multivariate Cox regression and least absolute shrinkage and selection operator (LASSO) regression analyses. The independent prognostic value of the risk model and the correlations of the prognostic signature with immune score, tumor immune cell infiltration, and immune checkpoints were systematically analyzed.

**Results:**

A prognostic risk model of four AGs (*BAK1*, *DKK1*, *CDKN2A*, and *MIF*) was constructed and validated in the training and testing datasets. Kaplan–Meier curves and time-dependent receiver operating characteristic (ROC) curve analysis confirmed that the four-AG risk signature possessed an accurate predictive value for the prognosis of patients with HNSCC. Correlation analysis revealed that the risk score was negatively associated with immune score and immune cell infiltration level while positively correlated with immune checkpoint blockade (ICB) response score. Patients of the high-risk subtype contained higher infiltration levels of resting natural killer (NK) cells, M0 macrophages, M2 macrophages, and resting mast cells while having lower infiltration levels of memory B cells, CD8+ T cells, follicular helper T cells, regulatory T cells (Tregs), and activated mast cells than did those of the low-risk subtype. The expressions of *CTLA4*, *PDCD1*, and *TIGIT* were downregulated while the *PDCD1LG2* expression was upregulated in the high-risk subtype compared to those in the low-risk subtype. Furthermore, the four selected AGs in the risk model were demonstrated to possess important functions in immune cell infiltration and ICB response of HNSCC.

**Conclusions:**

The aging-related risk signature is a reliable prognostic model for predicting the survival of HNSCC patients and provides potential targets for improving outcomes of immunotherapy.

## Introduction

Head and neck squamous cell carcinoma (HNSCC) is a frequent malignant tumor derived from the mucosal epithelium in the oral cavity, pharynx, and larynx, which presents an incidence rate of approximately 600,000 new cases and 350,000 deaths annually worldwide and an increasing prevalence among older adults (1, 2). In order to establish etiological risk factors related to lethal malignancy of HNSCC, in-depth studies have been conducted to analyze the genetic, epigenetic, and environmental factors that may trigger the occurrence of tumor (3). However, no effective screening strategy for HNSCC has been found so far, and careful physical examination is still the main method for early detection. In addition, most patients with advanced HNSCC present no clinical history of precancerous lesions (3). Therefore, there is an urgent need to identify novel biomarkers that are able to predict the progression of HNSCC precancerous lesions, treatment response, and survival.

Substantial efforts are being made to identify biomarkers for prejudging the prognosis of HNSCC patients. For example, the CXC chemokines have been proven to provide functional prognosis and suitable therapeutic value for HNSCC (4). A research cohort of 383 HNSCC patients revealed that elevated expression of NR2F6 is related to poorer recurrence-free survival, which could be used as a new prognostic indicator for early detection of local recurrences in patients with HNSCC (5). Recently, increasing autophagy- and ferroptosis-related risk signatures have been constructed to prognosticate survival for patients with HNSCC by a set of bioinformatics analyses (6–8). However, accurate and reliable prognostic indicators or models are still insufficient to improve the clinical outcomes of HNSCC patients.

The tumor microenvironment (TME) refers to a complex ecosystem involving persistent complex interactions between cancer cells, immune cells, stromal cells, and extracellular matrix components, which can play a significant role in tumor initiation and metastasis (9, 10). Substantial evidence has shown that a deep understanding of the heterogeneity of the TME within each type of cancer is essential to identify predictive biomarkers of patient outcomes that can routinely be used in the clinic (11, 12). Numerous reviews of published datasets have shown that the aging microenvironment can dramatically affect normal cells of the TME, which may have a huge impact on tumor progression (13–15). As one of the hallmarks of aging, inflammaging can result in degeneration of tissue and destruction of acute inflammation, which is closely correlated with the occurrence and progression of cancer (16, 17). Cellular senescence plays an important role in promoting the aging process and has been recognized as a primary factor that links inflammaging to a variety of age-related malignant tumors (17, 18). Age-related accumulation of senescence-associated secretory phenotype (SASP) cells can promote cancer progression by remaking the primary and metastatic microenvironment over time to a state where malignant cells are more likely to grow. Previous results have shown that cellular senescence is regulated by aging-related genes (AGs), which present important functions in tumor malignancy (19, 20). Given the tremendous changes in the extracellular matrix, secreted factors, and immune system as age increases, there may be potential clinical implications for immunotherapy and targeted therapy in patients with HNSCC. However, there is limited knowledge about the comprehensive correlations of the AGs with the tumor immune microenvironment (TIME) and the prognosis of HNSCC patients.

In this study, a risk model of the four AGs was constructed and was recognized as an independent prognostic index for HNSCC patients. The correlations of the risk model with immune score, immune cell infiltration, and potential immune checkpoints were systematically assessed based on the aging-related signatures. Furthermore, the effect of the four selected AGs on the TIME was explored to reveal their potential functions in tumor progression. Our findings contributed to clarify the regulatory mechanisms of the AGs related to the TIME and implied that these AGs might be functional prognostic biomarkers for predicting clinical outcomes of HNSCC patients and provide new targets for improving the immunotherapy response.

## Materials and Methods

### Data Sources and Processing

The fragments per kilobyte of exon model per million mapped reads (FPKM) RNA-sequencing data TCGA-HNSC (The Cancer Genome Atlas -Head and Neck Squamous Cell Carcinoma) and corresponding clinical follow-up information of HNSCC samples were obtained from TCGA data portal (https://portal.gdc.cancer.gov/). Aging-related human ontology gene sets were downloaded from the Molecular Signatures Database (MSigDB) (http://www.gsea-msigdb.org/gsea/msigdb/index.jsp) *via* searching by aging. These raw datasets of TCGA-HNSC were normalized by the multi-array average method, and the ENSEMBL gene ID was transformed to the GeneSymbol using the “biomaRt” package. The expressions of the same GeneSymbol were combined and unidentified gene IDs were excluded, whereas the expressions of 39,743 genes were analyzed.

### Identification of Prognostic Aging-Related Genes

The enrichment of aging-related gene sets was identified *via* the gene set enrichment analysis (GSEA, version 4.1.0) using TCGA-HNSC as expression datasets and aging-related human ontology gene sets as database. Nominal (NOM) P-values <0.05, |Normalized enrichment score (NES)| >1, and false discovery rate (FDR) q < 0.25 were used as filtering criteria. Then, the core AGs were obtained by screening the significantly upregulated AG sets in tumors from the GSEA *via* leading edge analysis. Finally, differentially expressed AGs between HNSCC and non-tumor samples were obtained using “Limma” package by R software (version 4.1.1) according to the cutoff criterion of |log2(fold change)| > 1 and *p* < 0.05.

These differentially expressed AGs were analyzed using univariate Cox regression to identify prognostic candidates (p < 0.05). Then, false-positive prognostic-associated AGs were eliminated by the least absolute shrinkage and selection operator (LASSO) Cox regression analyses. To improve the effectiveness and accuracy of prognostic prediction of these AGs, prognosis-related AGs were further analyzed by multivariate Cox regression to exclude those genes that cannot be used as independent indicators for prognostic monitoring.

### Construction and Validation of Aging-Related Gene-Related Prognostic Model

A risk model was established using the LASSO Cox regression analysis of prognosis-related AGs based on the training cohort (the whole dataset, n = 502). The risk score for each patient in both the training cohort and the testing cohort (randomly selected, n = 250) was estimated based on the formula, and patients were classified into high-/low-risk subtypes stratified by the median risk score. Kaplan–Meier analyses and log-rank test were conducted to compare survival differences between patients in the high-risk and low-risk subtypes. The time-dependent receiver operating characteristic (ROC) curves and corresponding areas under the curve (AUC) values were utilized to assess the prognostic value of AG-related risk model based on training and testing cohorts. Univariate and multivariate Cox regression analyses were conducted to further confirm the prognostic capacity of the AG-related signature. A nomogram and calibration plots were then built to further provide a prognosis of HNSCC that integrated clinicopathological factors. The relationships between the risk score with the overall survival (OS) and clinical characteristics of HNSCC patients were explored by stratification survival analyses.

### Determination of Associations of the Prognostic Model With Immune Cell Infiltration and Immune Checkpoint Genes

The immune scores of HNSCC samples were calculated with R package ESTIMATE (21). The infiltration levels of 22 types of immune cells of HNSCC samples were calculated *via* CIBERSORT (http://cibersort.stanford.edu/) (22). Then, the effect of the selected AGs in the risk model on infiltration levels of six types of immune cells was further analyzed *via* the TIMER database (https://cistrome.shinyapps.io/timer/) (23). To predict the immune status and potential immune checkpoint blockade (ICB) response, the correlations of the prognostic model with immune checkpoint expression and ICB response were investigated based on TCGA datasets. Potential ICB response was predicted with tumor immune dysfunction and exclusion (TIDE) algorithm (24).

### Statistical Analyses

The statistical analyses were carried out using R software (version 4.0.2) and GraphPad Prism 8 (San Diego, CA, USA) (25). Kaplan–Meier analyses were performed to compare OS between pairs of subtypes. The differences in the risk score between different clinical characteristic subtypes and differences in the immune cell infiltration level and immune checkpoints between the high and low subtypes stratified by the median risk score and median expression levels of *BAK1*, *DKK1*, *CDKN2A*, and *MIF* were evaluated by Wilcoxon test.

## Results

### Identification and Extraction of Differentially Expressed Aging-Related Genes in Head and Neck Squamous Cell Carcinoma

**Figure 1:**
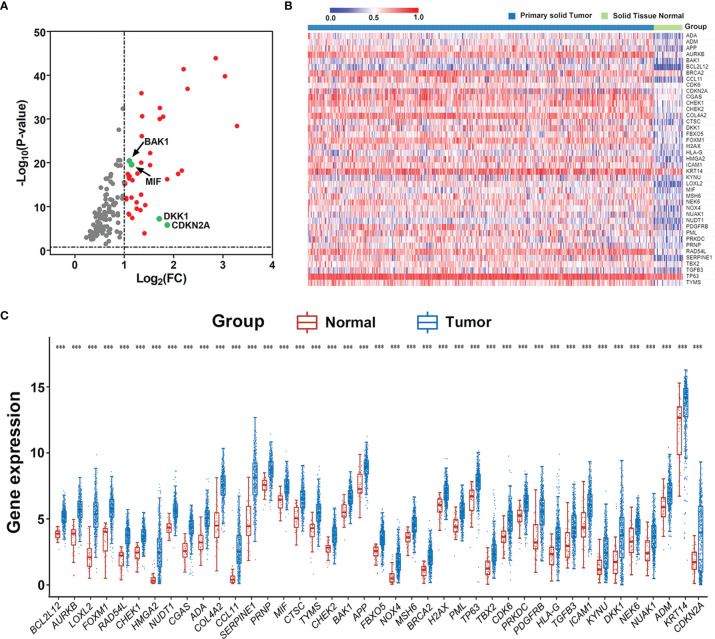
Extraction of differentially expressed aging-related genes (AGs) based on TCGA datasets. **(A)** Volcano plot for the 134 AGs in HNSCC retrieved from GSEA based on TCGA datasets. FC represents fold change. Red indicates upregulation, gray indicates no difference, and the studied four genes are marked in green. **(B)** Heat map of the 41 differentially expressed AGs in HNSCC. **(C)** Expression distribution of differentially expressed AGs in HNSCC and normal tissue samples. The red dot represents normal tissue sample, and the blue dot represents tumor sample. ***p < 0.001.

The RNA-sequencing data and clinical follow-up data of 502 HNSCC and 44 non-tumor samples were obtained from TCGA portal, and 11 aging-related human ontology gene sets were obtained from MSigDB. GSEA showed that 7 gene sets including “GOBP_CELL_AGING", “GOBP_REGULATION_OF_CELL_AGING", “GOBP_CELLULAR_ SENESCENCE", “GOBP_NEGATIVE_REGULATION_OF_CELL_AGING", “GOBP_POSITIVE_ REGULATION_OF_CELL_AGING", “GOBP_AGING”, and "GOBP_REPLICATIVE_SENESCENCE" are significantly enriched in HNSCC samples (**Figure S1**). A total of 134 core enrichment genes were identified as AGs *via* leading edge analysis of the above 7 gene sets (**Table S1**). Then, 41 significantly upregulated AGs were extracted according to the expression of all core enrichment genes in tumor and non-tumor samples (**Figures 1A, B** **)**. The differences in expression patterns of these 41 differentially expressed AGs were further presented in box plots (**Figure 1C**).

### Identification of Prognostic Risk Model of Aging-Related Genes in Head and Neck Squamous Cell Carcinoma

To investigate the prognostic value of differentially expressed AGs in HNSCC patients, univariate Cox proportional hazards regression analyses were conducted to estimate the prognostic relationship between AGs and OS in patients with HNSCC according to their mRNA expression level in the whole TCGA dataset (n = 502).

**Figure 2:**
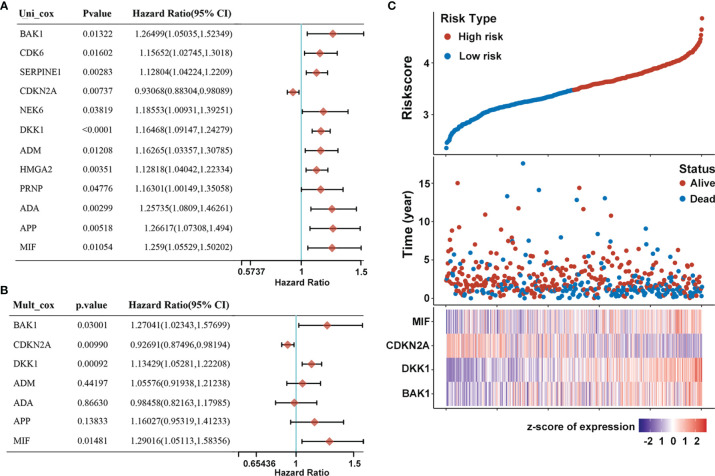
Construction of prognostic risk model of AGs for HNSCC patients. **(A)** Forest plot of 12 AGs that related to overall survival *via* univariate Cox regression analyses. **(B)** Multivariate Cox regression analyses of 7 prognosis-associated AGs. **(C)** Risk plot distribution, survival status of patients, and heat map of expression of included genes in the whole TCGA-HNSC dataset.

The prognostic value of differentially expressed AGs in HNSCC patients was explored using univariate Cox proportional hazards regression analyses based on the whole TCGA dataset (n = 502). Results showed that 12 differentially expressed AGs (*DKK1*, *SERPINE1*, *ADA*, *HMGA2*, *APP*, *CDKN2A*, *MIF*, *NEK6*, *ADM*, *BAK1*, *CDK6*, and *PRNP*) were remarkably related to the OS of HNSCC patients (*p* < 0.05, **Figure 2A**). Subsequently, false-positive prognosis-associated AGs were eliminated by LASSO Cox regression analyses, and 7 AGs including *BAK1*, *DKK1*, *CDKN2A*, *ADA*, *APP*, *MIF*, and *ADM* were obtained (**Figure S2**). Then, the impact of these 7 prognosis-associated AGs on OS and clinical outcomes of HNSCC patients was evaluated *via* multivariate Cox regression analyses (*p* < 0.05, **Figure 2B**). Finally, 4 AGs including *BAK1*, *DKK1*, *MIF*, and *CDKN2A* were identified with independent prognostic values for OS prediction and further applied to establish a prognostic risk model.

A prognostic risk model derived from the four selected AGs was constructed by LASSO Cox regression analyses, of which *BAK1*, *DKK1*, and *MIF* were identified as high-risk genes while *CDKN2A* was identified as a low-risk gene. The risk score formula was as follows: Risk score = (0.2126) * *BAK1* + (0.137) * *DKK1* + (-0.0716) * *CDKN2A* + (0.2474) * *MIF*. Afterward, patients were divided into high-risk and low-risk subtypes by the median risk score. The risk plot distribution and survival status of HNSCC patients showed that the OS rates of patients in the high-risk subtype were markedly lower than those in the low-risk subtype (**Figure 2C**). In addition, the heat map of the included genes showed that the expressions of *BAK1*, *DKK1*, and *MIF* were significantly higher in the high-risk subtype while the expression of *CDKN2A* was significantly higher in the low-risk subtype (**Figure 2C**).

**Figure 3:**
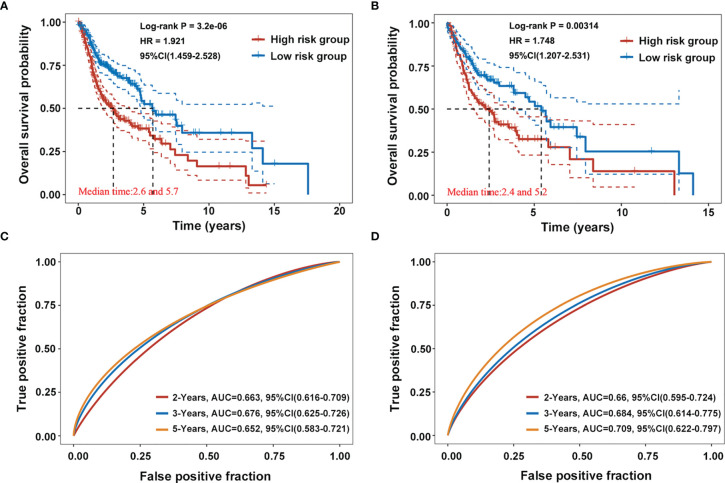
Validation of the prognostic capacity of the selected four-AG signature. **(A, B)** Kaplan–Meier survival curves for the risk model based on the training cohort (the whole dataset, n = 502) and the testing cohort (randomly selected, n = 250). **(C, D)** Receiver operating characteristic (ROC) curves for the risk model in the training and testing cohorts.

The prognostic value of the AG signature was validated in the training cohort (the whole dataset, n = 502) and the testing cohort (randomly selected, n = 250). Kaplan–Meier survival analyses showed that the high-risk group patients had a shorter OS than those of the low-risk group both in the training and testing cohorts (*p <* 0.001; **Figures 3A, B** **)**. The 2-, 3-, and 5-year AUC values of ROC curves for the AG signature in the training cohort were 0.663, 0.676, and 0.652, respectively (**Figure 3C**). And the corresponding 2-, 3-, and 5-year AUC values of ROC curves in the testing cohort were 0.660, 0.684, and 0.709, respectively (**Figure 3D**). These data together revealed the accuracy of the risk model of the four selected AGs for HNSCC prognosis.

### Associations of the Risk Model With Overall Survival and Clinicopathological Characteristics of Patients With Head and Neck Squamous Cell Carcinoma

**Figure 4:**
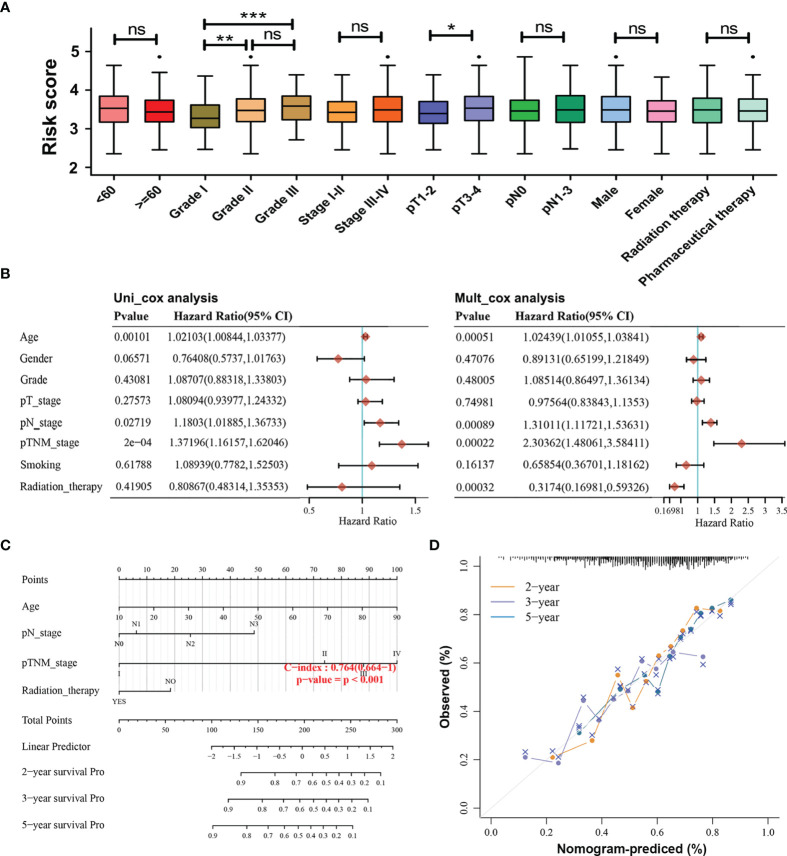
Associations of the risk model with the overall survival and clinicopathological characteristics of HNSCC patients. **(A)** Relationships between the risk score and clinicopathological characteristics of HNSCC patients. (ns) Non-significant, *p < 0.05, **p < 0.01, and ***p < 0.001. **(B)** Univariate and multivariate Cox analyses of clinical characteristics based on the training cohort. **(C)** The nomogram based on age, pathological N stage, pathological TNM stage, and radiation therapy for providing prognosis of HNSCC patients. **(D)** Calibration plots for the overall survival nomogram model. A dashed diagonal indicates the ideal nomogram, and the orange line, purple line, and blue line represent the predicted 2-year, 3-year, and 5-year overall survival of HNSCC patients.

To examine the associations of the risk model with clinicopathological characteristics of HNSCC patients, the differences in risk scores between subgroups sorted by age, tumor grade, pathological TNM stage, pathological T stage, pathological N stage, gender, and therapy were compared *via* Wilcoxon test. It was found that risk scores were significantly correlated with the tumor grade (*p* < 0.01) and pathological T stage (*p* < 0.05), whereas they showed no relationship with age, pathological TNM stage, pathological N stage, gender, and therapy (**Figure 4A**). Furthermore, univariate Cox regression analyses showed that the age, pathological N stage, and pathological TNM stage were significantly associated with OS in patients with HNSCC (*p <* 0.05, **Figure 4B**). Multivariate Cox regression analyses revealed that age, pathological N stage, pathological TNM stage, and radiation therapy were obviously related to OS in HNSCC patients (*p <* 0.05, **Figure 4B**). In order to provide a prognosis of HNSCC that integrated age, pathological N stage, pathological TNM stage, and radiation therapy, a nomogram with the C-index for survival prediction of 0.764 (*p* < 0.001) was built (**Figure 4C**). Calibration curves for the 2-, 3-, and 5-year OS nomogram model showed a good consistency between nomogram predictions and actual observations (**Figure 4D**).

**Figure 5:**
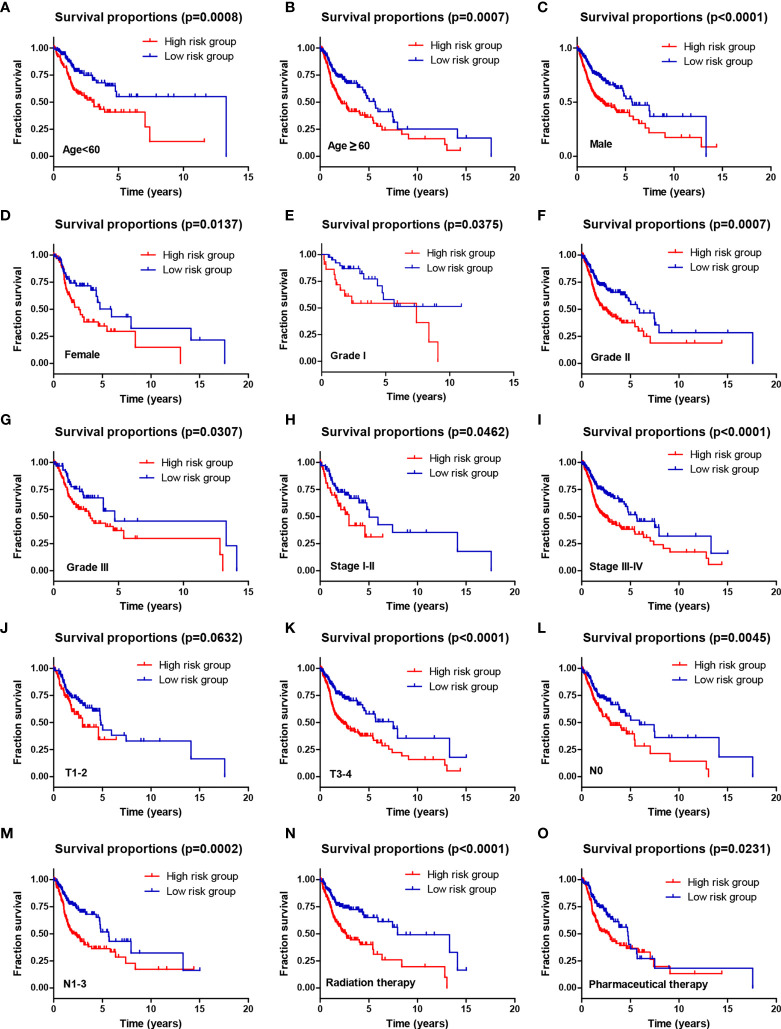
The prognostic ability of the four-AG signature for overall survival in multiple HNSCC subtypes. Kaplan–Meier curves for OS prediction in HNSCC subtypes of **(A)** Age <60 years, **(B)** Age ≥60 years, **(C)** Men, **(D)** Women, **(E)** Grade I, **(F)** Grade II, **(G)** Grade III, **(H)** Stages I–II, **(I)** Stages III–IV, **(J)** T1–2, **(K)** T3–4, **(L)** N0, **(M)** N1–3, **(N)** Radiation therapy, **(O)** Pharmaceutical therapy.

Moreover, stratification survival analyses were performed to assess the predictive ability of the four-AG risk model for prognosis in multiple HNSCC subtypes sorted by age (<60 years and ≥60 years), gender (Men and Women), tumor grade (Grade I, Grade II, and Grade III), pathological TNM stage (Stages I–II and Stages III–IV), pathological T stage (T1–2 and T3–4), pathological N stage (N0 and N1–2), and therapy (Radiation therapy and Pharmaceutical therapy) (**Figure 5**). Comparison of survival curves conducted by log-rank (Mantel–Cox) test revealed that patients of the high-risk group presented a remarkably poorer OS than that of the low-risk group in all HNSCC subtypes (all *p* < 0.05; **Figures 5A–I, K–O**) except pathological T1–2 HNSCC subtype (*p* > 0.05, **Figure 5J**). These results suggested that the identified four-AG risk model has a reliable predictive value for the prognosis of HNSCC.

### Associations of the Risk Model With Immune Score and the Immune Cell Infiltration Level in Head and Neck Squamous Cell Carcinoma

**Figure 6:**
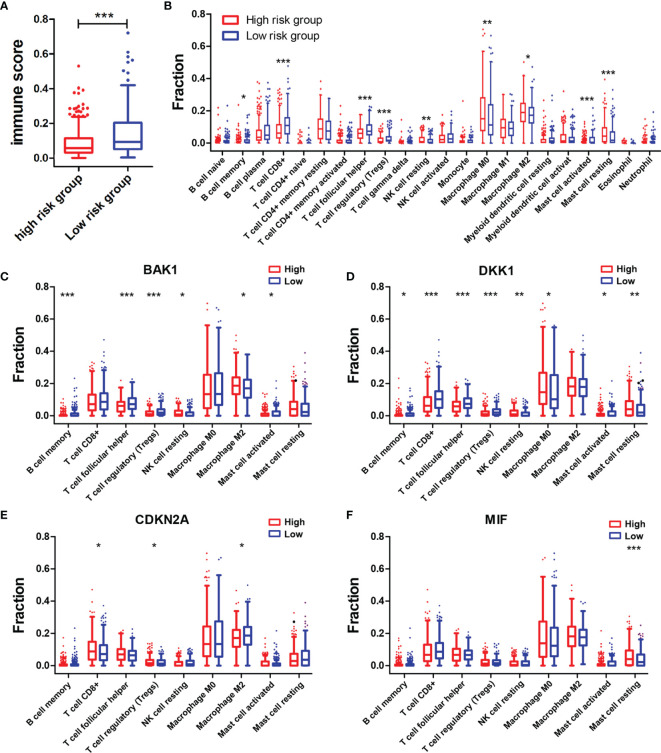
Associations of immune cell infiltration level with the risk score and the four selected AGs. **(A)** Comparison of immune scores in the high-risk and low-risk groups. **(B)** Comparison of compositional fractions of 22 types of immune cells between the high-risk and low-risk groups evaluated using the CIBERSORT formula. **(C–F)** Comparison of infiltration levels of nine types of immune cells according to *BAK1* **(C)**, *DKK1* **(D)**, *CDKN2A* **(E)**, and *MIF* **(F)** expression levels. *p < 0.05, **p < 0.01, and ***p < 0.001.

The association between the immune score and risk score was first investigated. The immune score in the high-risk group was notably lower than that in the low-risk group (*p* < 0.001) (**Figure 6A**). Subsequently, the differences in infiltration levels of 22 types of immune cells between high-risk and low-risk groups were evaluated using CIBERSORT formula (**Figure 6B**). Patients with HNSCC in the high-risk group contained lower infiltration levels of memory B cells (*p* < 0.05), CD8+ T cells (*p* < 0.001), follicular helper T cells (*p* < 0.001), regulatory T cells (Tregs) (*p* < 0.001), and activated mast cells (*p* < 0.001) while having higher infiltration levels of resting NK cells (*p* < 0.01), M0 macrophages (*p* < 0.01), M2 macrophages (*p* < 0.05), and resting mast cells (*p* < 0.001) than did those in the low-risk group. Then, the associations of the above nine types of immune cells with the selected AGs in the risk model were explored. HNSCC patients in the subtype of high *BAK1* expression included lower infiltration levels of memory B cells (*p* < 0.001), follicular helper T cells (*p* < 0.001), Tregs (*p* < 0.001), and activated mast cells (*p* < 0.05) while having higher infiltration levels of resting NK cells (*p* < 0.05) and M2 macrophages (*p* < 0.05) (**Figure 6C**). HNSCC patients in the subtype of high *DKK1* expression presented lower infiltration levels of memory B cells (*p* < 0.05), CD8+ T cells (*p* < 0.001), follicular helper T cells (*p* < 0.001), Tregs (*p* < 0.001), and activated mast cells (*p* < 0.05) while having higher infiltration levels of resting NK cells (*p* < 0.01), M0 macrophages (*p* < 0.05), and resting mast cells (*p* < 0.01) (**Figure 6D**). The results of *CDKN2A*, as a protective factor, showed that HNSCC patients with a high *CDKN2A* expression contained higher infiltration levels of CD8+ T cells (*p* < 0.05) and Tregs (*p* < 0.05) while having lower infiltration levels of M2 macrophages (*p* < 0.05) (**Figure 6E**). The results of *MIF* showed that only resting mast cells (*p* < 0.001) was positively associated with the expression of *MIF* (**Figure 6F**).

**Figure 7:**
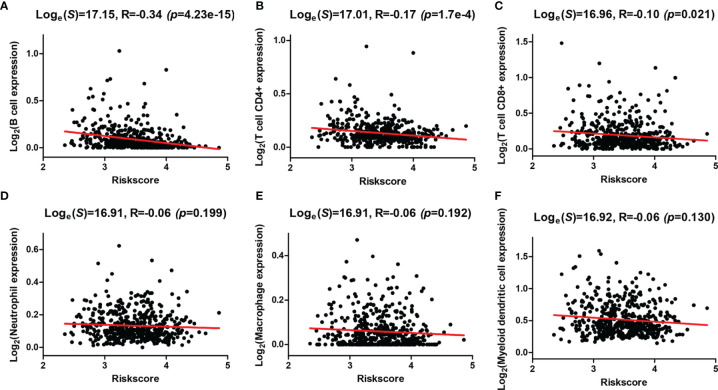
Correlations between the risk model and infiltration abundances of six types of immune cells. **(A–F)** Correlations between the risk score and six types of tumor-infiltrating immune cells including B cell **(A)**, T-cell CD4+ **(B)**, T cell CD8+ **(C)**, neutrophil **(D)**, macrophage **(E)**, and myeloid dendritic cell **(F)**.

In addition, correlation analysis of the risk score and infiltration levels of six types of immune cells was conducted based on the TIMER database to estimate the effect of the four-AG risk model on the TIME for HNSCC patients (**Figure 7**). Results showed that the risk score was negatively related to the infiltration of B cells (*p* < 0.001, **Figure 7A**), CD4+ T cells (*p* < 0.001, **Figure 7B**), and CD8+ T cells (*p* < 0.05, **Figure 7C**), whereas no significantly negative correlation was found between the risk score and infiltration of neutrophils (*p* > 0.05, **Figure 7D**), macrophages (*p* > 0.05, **Figure 7E**), and myeloid dendritic cells (*p* > 0.05, **Figure 7F**).

**Figure 8:**
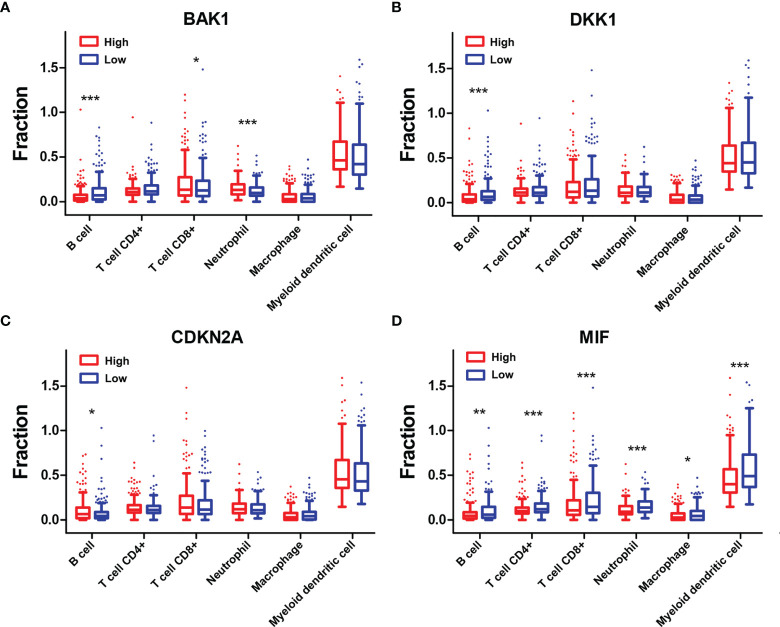
Association of six types of immune cells with the four selected AGs. **(A–D)** Comparison of six types of immune cells according to *BAK1* **(A)**, *DKK1* **(B)**, *CDKN2A* **(C)**, and *MIF* **(D)** expression levels. *p < 0.05, **p < 0.01, and ***p < 0.001.

To explore the mechanisms underlying AGs on the TIME in HNSCC, the relationships between the six types of immune cells and the four selected AGs were further investigated (**Figure 8**). HNSCC patients in the subtype of high *BAK1* expression included significantly higher infiltration levels of CD8+ T cells (*p* < 0.05) and neutrophils (*p* < 0.001) while having a lower infiltration level of B cells (*p* < 0.001) than did those in the subtype of low *BAK1* expression (**Figure 8A**). Only B cells were negatively related to the expression level of *DKK1* (*p <*0.001, **Figure 8B**). But for *CDKN2A*, as a protective factor, only B cells were positively related to the expression level of *CDKN2A* (*p* < 0.05, **Figure 8C**). HNSCC patients of the high-*MIF* expression subtype showed a lower infiltration level of B cells, CD4+ T cells, CD8+ T cells, neutrophils, macrophages, and myeloid dendritic cells than did those of low-*MIF* expression subtype (all *p* < 0.05, **Figure 8D**). The four selected AGs in the risk model (*BAK1*, *DKK1*, *CDKN2A*, and *MIF*) were demonstrated to possess important functions in regulating the TIME of HNSCC.

### Association of Immune Checkpoints and Immune Checkpoint Blockade Response Scores With the Risk Score and the Four Selected Aging-Related Genes

**Figure 9:**
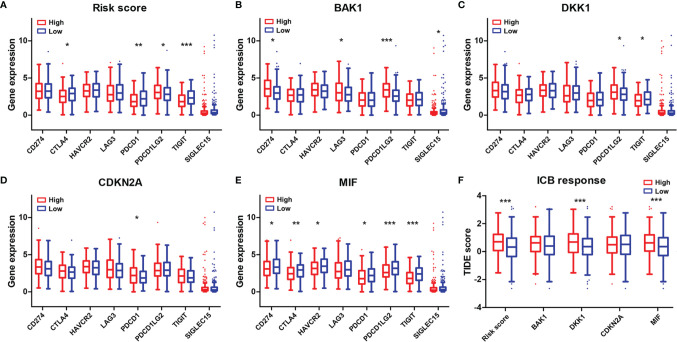
Association of the four-AG signature with immune checkpoints and ICB response scores. **(A–E)** Comparison of eight immune checkpoint genes in the high and low subtypes stratified by risk score **(A)** and expression levels of *BAK1* **(B)**, *DKK1* **(C)**, *CDKN2A* **(D)**, and *MIF* **(E)**. **(F)** Comparison of ICB response scores according to risk score **(A)** and expression levels of *BAK1*, *DKK1*, *CDKN2A*, and *MIF*. *p < 0.05, **p < 0.01, and ***p < 0.001.

The expression levels of eight immune checkpoint genes including *CD274*, *CTLA4*, *HAVCR2*, *LAG3*, *PDCD1*, *PDCD1LG2*, *TIGIT*, and *SIGLEC15* were calculated to explore the correlations of immune checkpoints with the risk score and the four selected AGs. The expressions of *CTLA4*, *PDCD1*, and *TIGIT* were found to be downregulated while *PDCD1LG2* expression was found to be upregulated in the high-risk subtype (**Figure 9A**). *BAK1* showed crucially positive correlations with the expression of *CD274*, *LAG3*, and *PDCD1LG2* while having a negative correlation with *SIGLEC15* expression (**Figure 9B**). *DKK1* showed a positive relationship with the expression of *PDCD1LG2* while having a negative relationship with the expression of *TIGIT* (*p* < 0.05, **Figure 9C**). *CDKN2A* revealed a positive correlation with *PDCD1* expression (*p* < 0.05, **Figure 9D**). *MIF* revealed significantly negative correlations with the expressions of *CD274*, *CTLA4*, *HAVCR2*, *PDCD1*, *PDCD1LG2*, and *TIGIT* (*p* < 0.05, **Figure 9E**). Furthermore, the differences of ICB response scores in the high and low subtypes stratified by risk score and the four selected AGs were evaluated. Results revealed that the ICB response scores were crucially higher in the high-risk subtype, high-*DKK1* expression subtype, and high-*MIF* expression subtype than those in the low subtypes (*p* < 0.001, **Figure 9F**).

## Discussion

The quality of life and life expectancy for HNSCC patients have been significantly improved by advances in robotic surgery, radiation and chemotherapy, immunotherapy, and molecular characterization of human cancers (3). However, the high mortality rate and outcomes of patients with advanced-stage HNSCC have remained mostly unchanged due to the lack of effective and reliable predictive biomarkers for monitoring HNSCC development (1, 3). Thus, the identification of novel disease-related prognostic biomarkers with adequate performance and clinical convenience in HNSCC is necessary to develop novel therapeutic approaches for improving treatment outcomes.

Studies have highlighted that many of the hallmarks of aging are the same as those of cancer, such as epigenetic changes, altered intracellular communication, changes in proteostasis, mitochondrial dysfunction, and cellular senescence (26). Some of these shared common characteristics, including increased accumulation of genomic damage, telomere attrition, epigenetic alteration, impaired proteostasis, and nutritional perception disorders, may be attributed to tumor progression (27–29). It has been demonstrated that age-dependent changes in inflammation and immune cell infiltration levels played important roles in tumor progression and malignancy (30). Therefore, systematically exploring the functions of AGs in the TIME is necessary to identify the function of the aging microenvironment in HNSCC progression. However, few studies to date have explored the associations of AGs with the TIME and the OS of patients with HNSCC.

In this study, we identified 41 differentially expressed AGs by GSEA based on the training (TCGA) dataset and further constructed a four-AG risk signature using the LASSO Cox regression analysis. It was confirmed that the risk model of the four selected AGs could be recognized as a satisfactory independent prognostic indicator for predicting the clinical outcomes of HNSCC patients. *BAK1*, *DKK1*, and *MIF* acted as risk factors while *CDKN2A* acted as a protective factor in the risk model. *BAK1* played important roles in the mitochondrial apoptosis process, which could alter the permeability of the mitochondrial outer membrane to induce cell apoptosis (31). *DKK1* is an antagonist of Wnt signaling, which regulates various cellular and biological processes that play important roles in cell senescence, cell apoptosis, differentiation, and metastasis in various tissues and numerous cancers (32, 33). Previous studies had shown that elevated expression of *DKK1* was an independent adverse prognostic indicator of survival in HNSCC (34, 35). *MIF* has been determined as a pro-inflammatory cytokine that plays multiple roles in inflammation and angiogenesis and was associated with carcinogenesis (36). It had been reported that overexpression of *MIF* promoted tumor metastasis and was notably associated with poor prognosis of pancreatic ductal adenocarcinoma (37). Kindt etal. (38) demonstrated that the elevated expression of *MIF* resulted in tumor progression and poorer prognosis of HNSCC. For *CDKN2A*, it had been shown that methylation of *CDKN2A* was highly associated with HNSCC carcinogenesis and bad OS, which could be a diagnostic and prognostic indicator for HNSCC (39). In addition, it has been demonstrated that the copy number loss of *CDKN2A* could be an effective prognostic biomarker to independently predict poor survival in human papilloma virus (HPV)-negative HNSCC (40).

The clinic correlation analysis confirmed that the risk score was highly associated with tumor grade and pathological T stage. Notably, stratification survival analysis revealed that the four-AG risk signature possessed an accurate predictive value for prognosis in multiple HNSCC subtypes sorted by age, gender, tumor grade, pathological TNM stage, pathological T stage, pathological N stage, and therapy. Calibration curves for the 2-, 3-, and 5-year OS nomogram model showed good consistency between nomogram predictions and actual observations. Notably, the 2-, 3-, and 5-year AUC values of ROC curves for the four-AG signature in both the training and testing cohorts were higher than 0.65, which is better than those of previous prognostic signatures of the seven AGs for HNSCC (41). In addition, the total point of clinic efficacy in our nomogram is much higher than those of previously developed and validated models (8, 41). These results suggested that the identified four-AG risk model has a reliable predictive value for prognosis of HNSCC.

The TME in HNSCC harbors a complex interplay between tumor cells and stromal cells, including endothelial cells, cancer-associated fibroblasts (CAFs), and immune cells, which can promote HNSCC development (3). Numerous studies have reported that HNSCCs are highly infiltrated by immune cells, and different immunophenotypes combining several molecular features have been identified as classifiers for HNSCC, which might be useful in predicting response to different therapies, especially checkpoint inhibition (42, 43). Tumor-infiltrating lymphocytes [including T cells, B cells, and natural killer (NK) cells] and myeloid lineage cells (including macrophages, neutrophils, dendritic cells, and myeloid-derived suppressor cells) are the main immune components of the HNSCC TME (3). Previous studies showed that high levels of tumor-infiltrating lymphocytes were usually correlated with better prognosis of HNSCC depending on the balance of cells with antitumor activity vs. those with immunosuppressive activities (44, 45). Effector T cells and NK cells mainly regulated the antitumor immunity in the TME, whereas Tregs, myeloid dendritic cells, and M2 macrophages largely regulated the immune suppression and tumor cell growth. It had been proven that high infiltration levels of Tregs, myeloid dendritic cells, neutrophils, or M2 macrophages were related to advanced-stage HNSCC or bad OS, whereas high infiltration levels of CD8+ effector T cells and NK cells in the TME were related to better outcomes (46). In this study, patients of the high-risk group contained higher infiltration levels of resting NK cells, M0 macrophages, M2 macrophages, and resting mast cells while having lower infiltration levels of memory B cells, CD8+ T cells, follicular helper T cells, Tregs, and activated mast cells than did those of the low-risk group. In addition, negative correlations between the risk score and infiltration levels of the B cells, CD4+ T cells, CD8+ T cells, neutrophils, macrophages, and myeloid dendritic cells were observed based on TIMER database. All these data suggested that the integral index of the immunoscore obtained on the basis of the expression of the four AGs included in our prognostic signature and expression levels of these AGs are significantly correlated with immunosuppressive activity for HNSCC patients. To explore the mechanisms of AGs on the TIME, the correlations between the four selected AGs and immune cell infiltration levels were investigated. It was found that patients of subtypes of high *BAK1* and *DKK1* expressions contained lower infiltration levels of memory B cells, CD8+ T cells, follicular helper T cells, Tregs, and activated mast cells, suggesting a low immunosuppressive activity for HNSCC patients of the high-expression groups. Based on the TIMER database, HNSCC patients in the subtype of high *BAK1* expression included significantly higher infiltration levels of CD8+ T cells and neutrophils while having a lower infiltration level of B cells. HNSCC patients of the high-*MIF* expression subtype showed a lower infiltration level of B cells, CD4+ T cells, CD8+ T cells, neutrophils, macrophages, and myeloid dendritic cells than did those of the low-*MIF* expression subtype. Results showed that the selected AGs in our risk model (*BAK1*, *DKK1*, and *MIF*) were negatively related to the infiltration levels of tumor-infiltrating lymphocytes (including T cells, B cells, and macrophages), suggesting a low immunosuppressive activity for HNSCC patients of the high-risk groups.

Furthermore, we illustrated the relationships of immune checkpoints with the risk score and the four selected AGs to predict the response to different therapies, particularly checkpoint inhibition. The immune checkpoint molecule is an inhibitory regulatory molecule in the immune system, which is essential for maintaining self-tolerance, preventing autoimmune response, and minimizing tissue damage by controlling the time and intensity of the immune response (47). Immune checkpoint molecules expressed on immune cells will inhibit the function of immune cells and prevent the body from producing effective antitumor immune responses, which can result in tumor immune escape (48). Thus, the decreased expression of immune checkpoints may prevent tumor immune escape to improve the outcomes of immunotherapy. Our results showed that the expressions of *CTLA4*, *PDCD1*, and *TIGIT* were found to be downregulated in the high-risk subtype; *BAK1* showed crucially positive correlations with the expressions of *CD274*, *LAG3*, and *PDCD1LG2* while having a negative correlation with *SIGLEC15* expression; *DKK1* showed a positive relationship with the expression of *PDCD1LG2* while having a negative relationship with the expression of *TIGIT*; *CDKN2A* revealed a positive correlation with *PDCD1* expression; and *MIF* revealed significantly negative correlations with the expressions of *CD274*, *CTLA4*, *HAVCR2*, *PDCD1*, *PDCD1LG2*, and *TIGIT*. Hence, the four selected AGs in our risk model (*BAK1*, *DKK1*, *CDKN2A*, and *MIF*) performed important functions on immune checkpoint expressions, which might be regarded as potential targets to improve the outcomes of immunotherapy by downregulating the expressions of immune checkpoints.

Moreover, the relationships of the ICB response score (TIDE score) with the risk score and the four selected AGs were evaluated. TIDE uses a set of gene expression markers to evaluate two different tumor immune escape mechanisms, including the dysfunction of tumor-infiltrating cytotoxic T lymphocytes (CTLs) and the rejection of CTLs by immunosuppressive factors (24). The high ICB response score indicates the poor efficacy of the immune checkpoint-blocking therapy (ICB) and the short survival period after receiving ICB treatment. Our results revealed that the ICB response scores were crucially higher in the high-risk subtype, high-*DKK1* expression subtype, and high-*MIF* expression subtype than those in the low subtypes, which suggest poor immunotherapy efficacy for patients with HNSCC in the high-risk groups.

Nevertheless, there are several limitations in this study. First, the risk signature of the four AGs was obtained and validated based on TCGA datasets. The prognostic value of the AG-related signature in HNSCC patients has not been externally validated due to the lack of our own adequate available data. External validation should be performed based on our own clinic data in the future. Second, the relationship between the risk signature and the immune cell infiltration level and the role of the AG-related signature in immunotherapy efficacy were revealed *via* comprehensive bioinformatics analysis. More *in vitro* and *in vivo* experiments are needed to confirm these conclusions. Laboratory experiments will be done in future research to validate the specific roles of the AG-related signature in immunotherapy efficacy for patients with HNSCC.

## Conclusions

In conclusion, our study constructed a risk signature of four AGs that could be utilized as an independent prognostic indicator for HNSCC patients and showed important functions in immunosuppression of HNSCC. Meanwhile, the AG-related signature might provide potential targets to improve the outcomes of immunotherapy.

## Data Availability Statement

The datasets presented in this study can be found in online repositories. The names of the repository/repositories and accession number(s) can be found in the article/**Supplementary Material**.

## Author Contributions

FC and JL contributed to conception and design of the study. FC, XG, and MX organized the database and performed the statistical analysis. FC wrote the first draft of the article. FY, JW, and CY wrote sections of the article. All authors contributed to article revision and read and approved the submitted version.

## Funding

This work was supported by grants from the Science and Technology Planning Project of Guangzhou (202102010041) and Health Science and Technology Project of Guangzhou (20211A010014).

## Conflict of Interest

The authors declare that the research was conducted in the absence of any commercial or financial relationships that could be construed as a potential conflict of interest.

## Publisher’s Note

All claims expressed in this article are solely those of the authors and do not necessarily represent those of their affiliated organizations, or those of the publisher, the editors and the reviewers. Any product that may be evaluated in this article, or claim that may be made by its manufacturer, is not guaranteed or endorsed by the publisher.
