# Supplementary figures and images for: The Aging-Related Prognostic Signature Reveals the Landscape of the Tumor Immune Microenvironment in Head and Neck Squamous Cell Carcinoma
Source: Front Oncol. 2022 May 10;12:857994. doi: 10.3389/fonc.2022.857994 (PMC9127417; doi:10.3389/fonc.2022.857994)

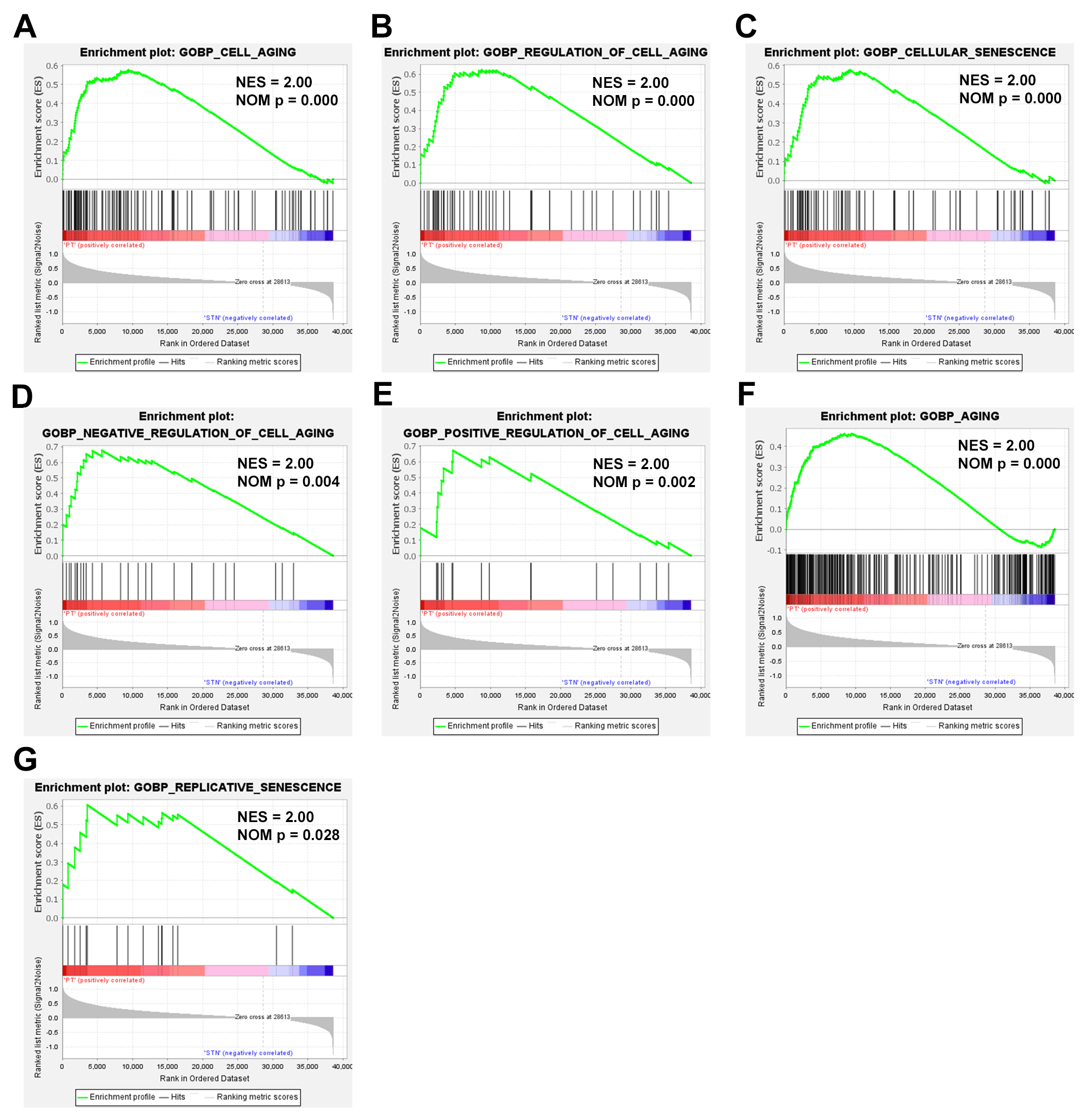

Supplement: Supplementary file 1 [file Image_1.tif]

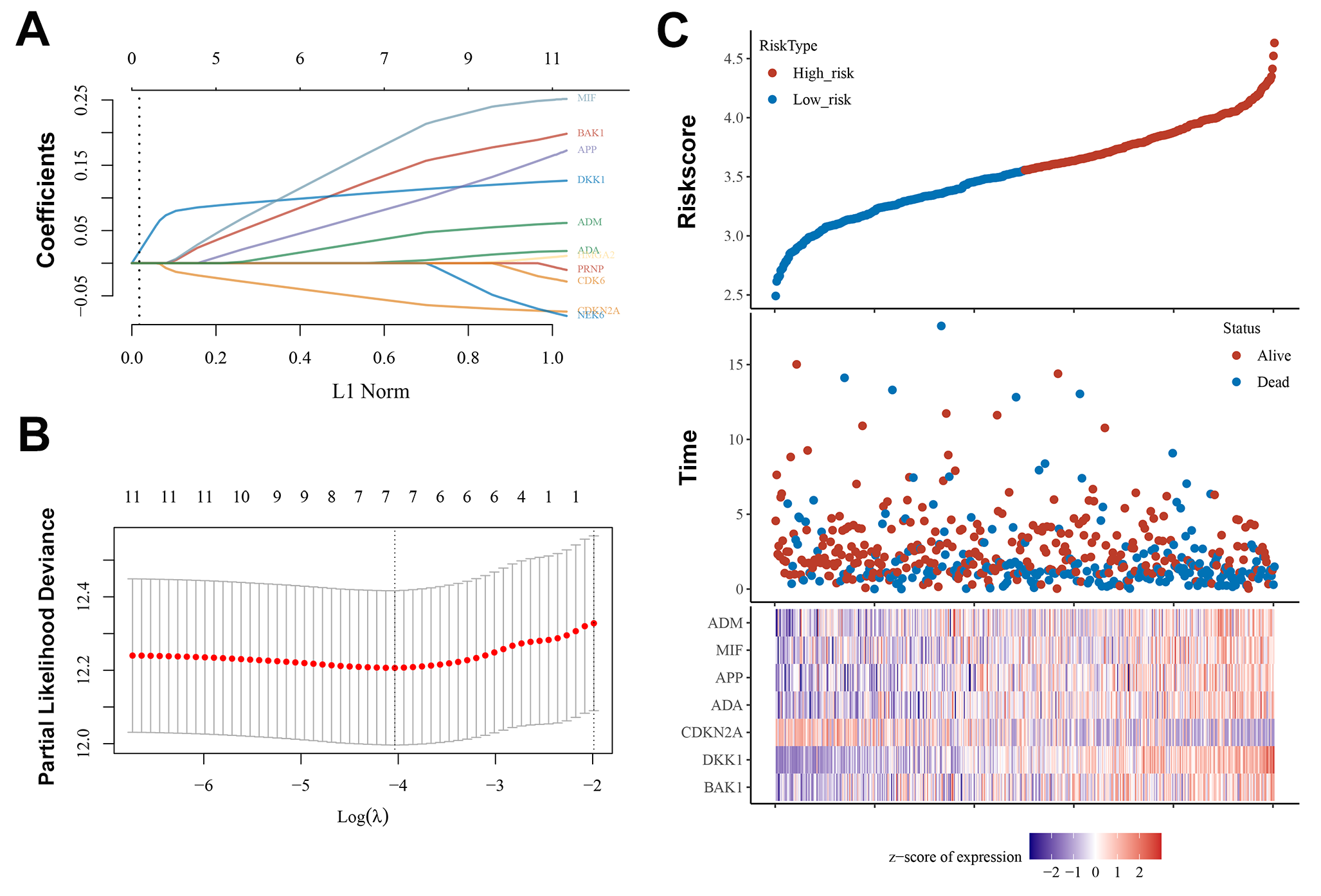

Supplement: Supplementary file 2 [file Image_2.tif]
